# ComMap: a software to perform large-scale structure-based mapping for cross-linking mass spectrometry

**DOI:** 10.1093/bioinformatics/btad077

**Published:** 2023-02-20

**Authors:** Weijie Zhang, Yichu Shan, Lili Zhao, Zhen Liang, Chao Liu, Lihua Zhang, Yukui Zhang

**Affiliations:** CAS Key Laboratory of Separation Science for Analytical Chemistry, National Chromatographic R. & A. Center, Dalian Institute of Chemical Physics, Chinese Academy of Sciences, Dalian, Liaoning 116023, China; University of Chinese Academy of Sciences, Beijing 100039, China; CAS Key Laboratory of Separation Science for Analytical Chemistry, National Chromatographic R. & A. Center, Dalian Institute of Chemical Physics, Chinese Academy of Sciences, Dalian, Liaoning 116023, China; CAS Key Laboratory of Separation Science for Analytical Chemistry, National Chromatographic R. & A. Center, Dalian Institute of Chemical Physics, Chinese Academy of Sciences, Dalian, Liaoning 116023, China; University of Chinese Academy of Sciences, Beijing 100039, China; CAS Key Laboratory of Separation Science for Analytical Chemistry, National Chromatographic R. & A. Center, Dalian Institute of Chemical Physics, Chinese Academy of Sciences, Dalian, Liaoning 116023, China; School of Engineering Medicine and School of Biological Science and Medical Engineering, Beihang University, Beijing 100191, China; CAS Key Laboratory of Separation Science for Analytical Chemistry, National Chromatographic R. & A. Center, Dalian Institute of Chemical Physics, Chinese Academy of Sciences, Dalian, Liaoning 116023, China; CAS Key Laboratory of Separation Science for Analytical Chemistry, National Chromatographic R. & A. Center, Dalian Institute of Chemical Physics, Chinese Academy of Sciences, Dalian, Liaoning 116023, China

## Abstract

**Motivation:**

Chemical cross-linking combined with mass spectrometry (CXMS) is now a well-established method for profiling existing protein–protein interactions (PPIs) with partially known structures. It is expected to map the results of CXMS with existing structure databases to study the protein dynamic profile in the structure analysis. However, currently available structure-based analysis software suffers from the difficulty of achieving large-scale analysis. Besides, it is infeasible for structure analysis and data mining on a large scale, since of lacking global measurement of dynamic structure mapping results.

**Results:**

ComMap (protein complex structure mapping) is a software designed to perform large-scale structure-based mapping by integrating CXMS data with existing structures. It allows complete the distance calculation of PPIs with existing structures in batch within minutes and provides scores for different PPI-structure pairs of testable hypothetical structural dynamism via a global view.

**Supplementary information:**

Supplementary data are available at *Bioinformatics* online.

## 1 Introduction

Chemical cross-linking combined with mass spectrometry (CXMS) has been widely applied in the research of protein interaction and structure in recent years (Wheat *et al.*, 2021). In CXMS, cross-linkers are used to covalently link two residues to form an informative linkage between proteins, which can be identified from MS/MS spectra by dedicated software (Yu and Huang, 2018). By mapping the cross-linking results to the existing protein structures, complete cross-linked protein structure information can be delineated and can be utilized for subsequent protein complexes structural analysis or protein docking analysis. However, current structure software can neither achieve the structural analysis on large scale nor measure the global dynamic structure mapping results. For instance, proXL (Riffle *et al.*, 2016) and xiView (Graham *et al.*, 2019) can only load one single user-defined Protein Data Bank file at a time, while some other web programs designed for batch processing, such as XLinkDB are focused on structural mapping of specific Lys–Lys cross-linkers (Schweppe *et al.*, 2016) so that site-non-specific cross-linkers are deserted (Zhang *et al.*, 2022). Moreover, the results of CXMS involved dynamic information. ‘Over-length’ cross-links may arise from the alternative excited-state conformation of the protein complex (Ding *et al.*, 2017). Recently, a comprehensive structure-based evaluation of different search tools has been performed (Yugandhar *et al.*, 2020). Unfortunately, the above information is not considered in the current structure mapping tools. For this reason, researchers can only perform dynamic analysis for several proteins without a comparable scoring system, but not dynamism profile via a global view. Lastly, present protein interactions docking and structural simulation based on cross-linking information rely on manually filtered cross-links, without algorithmic discrimination, which may bring excessive subjective impact on the quality of model construction (Mintseris and Gygi, 2020). It’s infeasible for the CXMS community to analyze and perform structure-based data mining in biological systems rapidly on large scale.

Herein, we propose ComMap, a software designed to perform large-scale structure-based mapping on CXMS data. First, ComMap enables reading thousands of protein structure files and completing the distance calculation from existing structures in minutes. Second, ComMap is not restricted to specific cross-linking protocols and is capable of kinds of cross-linkers.

Moreover, ComMap identifies known static PPIs and measures testable hypothetical dynamism of protein structure across diverse PPI-structure pairs. Hence, biologists can now perform structural dynamic analysis and protein docking for thousands of PPIs under a comparable scoring standard.

## 2 Implementation

**Fig. 1.**
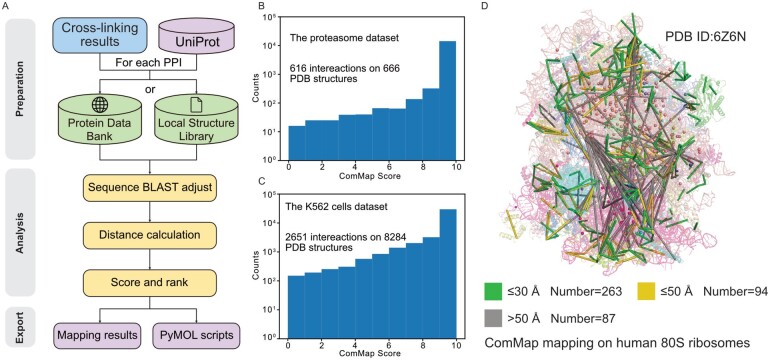
Computational workflow of ComMap and applications of ComMap on the proteasome dataset and the K562 cells dataset. (**A**) Algorithmic design of ComMap. (**B**) The ComMap scores distribution on the proteasome dataset. (**C**) The ComMap scores distribution on the K562 cells dataset. (**D**) An example of PPIs mapping on human 80S ribosomes with ComMap from the K562 cells dataset

ComMap is implemented in Python 3.8 and includes three steps: data preparation, distance analysis and results export (Fig. 1A).

### 2.1 Data preparation

Firstly, ComMap reads cross-linking results, which are generated from pLink2, SpotLink or generic PPI files, and the corresponding fasta file. Next, ComMap reads local or online Protein Data Bank (PDB) protein structure files in mmCIF format according to user configurations (detailed description in Supplementary Note S1).

### 2.2 Distance analysis

Next, ComMap performs alignment on the protein structure sequence and identified protein sequences using the BLAST tool. After that, ComMap calculates the residue distance for each PPI-structure pair. Following, ComMap scores the PPI-structure pairs based on several features, which reflects structural dynamism via a global view (detailed description in Supplementary Note S1).

### 2.3 Results export

Finally, ComMap generated three files as outputs, including a comprehensive file that saved all PPI and structural distance information, a categorized file of PPIs based on structure entries and a ComMap score file for PPI-structure pairs. Pymol scripts of protein structures can be easily achieved for visualization in these files.

## 3 Results

Three PPIs datasets were analyzed by ComMap as an illustration, including the BSA dataset, the proteasome dataset and the K562 cells dataset.

To demonstrate the ability of ComMap to analyze multiple types of cross-linkers generated data, we analyzed the BSA dataset at first (Iacobucci *et al.*, 2019), which involved DSS, BS3 and DSBU cross-linkers (detailed description in Supplementary Note S2). The ComMap scores distribution in this dataset concentrates in the high-scoring region, indicating relatively static interactions, which correspond to BSA characteristics. Besides, we offered a step-by-step user guide for ComMap on the BSA dataset.

To demonstrate the performance of ComMap score in target protein complexes, we obtained and analyzed the proteasome dataset from a sample of *Saccharomyces cerevisiae* (PXD011296) (Mintseris and Gygi, 2020). ComMap observed the distance information of 616 proteasome interactions from 666 protein structures and generated high-density proteasome PPI-structure mapping. Analysis of the ComMap score demonstrated that most interactions are relatively static interactions in this dataset (Fig. 1B). In-depth investigation of these interactions demonstrated that ComMap is a reliable tool for analyzing protein cross-linking and structural dynamics (detailed description in Supplementary Note S3).

Then ComMap was used to explore *Homo sapiens*-related PPIs. We obtained and analyzed the human cell cross-linking dataset from the K562 cells sample (PXD018771) (Yugandhar *et al.*, 2020). ComMap observed the distance information of 2651 interactions from 8284 protein structures. We observed ComMap score distributions on this dataset similar to those on the proteasome dataset (Fig. 1C). An example of the PPI mapping on human 80S ribosomes with ComMap from this dataset is illustrated (Fig. 1D, detailed structure information in Supplementary Materials). Additionally, we discovered testable hypothesis of dynamic property from several protein complexes, including ribosome, GTP-binding nuclear protein and calmodulin protein (detailed description in Supplementary Note S4).

## 4 Conclusions

As a result, ComMap is a valuable tool for structure-based dynamism measurement analysis on CXMS data ranging from target protein complex to proteome in a flexible and dynamic way. However, at present, all the cross-linking structure dynamism analysis can only be performed based on the recorded structures in PDB. Machine learning methods could be probably introduced in the scoring system in the future, which may reduce recorded structures dependency. With sufficient learning experience brought by cumulative datasets, ComMap will greatly facilitate biologists for protein structure and PPIs studies or CXMS-based protein docking studies.

## Supplementary Material

btad077_Supplementary_Data

## Data Availability

ComMap is available at https://github.com/DICP1810/ComMap.

## References

[btad077-B1] Ding Y.H. et al (2017) Modeling protein excited-state structures from “over-length” chemical cross-links. J. Biol. Chem., 292, 1187–1196. 27994050 10.1074/jbc.M116.761841 PMC5270465

[btad077-B2] Graham M. et al (2019) xiView: A common platform for the downstream analysis of crosslinking mass spectrometry data. bioRxiv 561829.

[btad077-B3] Iacobucci C. et al (2019) First community-wide, comparative cross-linking mass spectrometry study. Anal. Chem., 91, 6953–6961. 31045356 10.1021/acs.analchem.9b00658 PMC6625963

[btad077-B4] Mintseris J. , Gygi S.P. (2020) High-density chemical cross-linking for modeling protein interactions. Proc. Natl. Acad. Sci. USA, 117, 93–102. 31848235 10.1073/pnas.1902931116 PMC6955236

[btad077-B5] Riffle M. et al (2016) ProXL (protein cross-linking database): A platform for analysis, visualization, and sharing of protein cross-linking mass spectrometry data. J. Proteome Res., 15, 2863–2870. 27302480 10.1021/acs.jproteome.6b00274 PMC4977572

[btad077-B6] Schweppe D.K. et al (2016) XLinkDB 2.0: Integrated, large-scale structural analysis of protein crosslinking data. Bioinformatics, 32, 2716–2718. 27153666 10.1093/bioinformatics/btw232 PMC5013903

[btad077-B7] Wheat A. et al (2021) Protein interaction landscapes revealed by advanced in vivo cross-linking–mass spectrometry. Proc. Natl. Acad. Sci. USA, 118, e2023360118. 34349018 10.1073/pnas.2023360118 PMC8364181

[btad077-B8] Yu C. , Huang L. (2018) Cross-linking mass spectrometry: An emerging technology for interactomics and structural biology. Anal. Chem., 90, 144–165. 29160693 10.1021/acs.analchem.7b04431 PMC6022837

[btad077-B9] Yugandhar K. et al (2020) Structure-based validation can drastically underestimate error rate in proteome-wide cross-linking mass spectrometry studies. Nat. Methods, 17, 985–988. 32994567 10.1038/s41592-020-0959-9 PMC7534832

[btad077-B10] Zhang W. et al (2022) SpotLink enables sensitive and precise identification of site nonspecific cross-links at the proteome scale. Brief. Bioinform., bbac316 10.1093/bib/bbac316 36093786

