# Supplementary material for: ComMap: a software to perform large-scale structure-based mapping for cross-linking mass spectrometry
Source: Bioinformatics. 2023 Feb 20;39(2):btad077. doi: 10.1093/bioinformatics/btad077 (PMC9960907; doi:10.1093/bioinformatics/btad077)
Supplement: btad077_Supplementary_Data [file btad077_supplementary_data.zip › Supplementary Note.docx]

Supplementary Note 1. The workflow of ComMap

ComMap, coded in Python 3.8, making use of Numpy (Harris, et al., 2020), Pandas and lxml libraries, consisted of three steps, as shown in Figure 1A: data preparation (includes reading MS/MS identified protein-protein interactions (PPIs) information and obtaining structural information of proteins from the Internet or local files), distance analysis (includes performing protein sequence alignment, calculating PPIs residue distance, scoring and ranking) and results export (writing output files).

1. Data preparation

ComMap first read the fasta file used to identify cross-links and the identification results generated from the cross-linking software (such as pLink2(Chen, et al., 2019), SpotLink(Zhang, et al., 2022)). A generic tab-delimited file containing PPIs can also be used as input for ComMap. For additional information, please check the user manual.

Then, ComMap obtained the structure information of relevant proteins. In online mode, structural information of the protein was queried in the UniProt database and obtained in the Protein Data Bank (PDB) database. In offline mode, the information was read from local files. ComMap by default used CIF-format protein structure files, which can store extremely large protein structures. For additional information, please check the user manual.

1. Distance analysis

After that, ComMap performed sequence alignment between protein fasta sequences and protein structure sequences. This eliminated the mistakes caused by amino acid or sequence deletions and mutations in the distance calculation. ComMap utilized the NCBI-provided BLAST (Altschul, et al., 1990) tool for this part.

Next, ComMap calculated the residue distance for all PPI-structure pairs. Typically, the distance of Cα-Cα from residues was calculated.

Following, ComMap scored and ranked each PPI-structure pair, which measured structural dynamism information from the cross-linking samples. For this part, a comprehensive scoring function was designed for evaluation, based on the distance between residues from each PPI-structure pair. The function included three probability estimation factors and one offset factor.

1. $\boldsymbol{p}\left( \boldsymbol{dis=i,}\boldsymbol{PPI}_{\boldsymbol{j}} | \boldsymbol{S}_{\boldsymbol{i}} \right)$ and $\boldsymbol{p}\left( \boldsymbol{dis=i,}\boldsymbol{S}_{\boldsymbol{i}} | \boldsymbol{PPI}_{\boldsymbol{j}} \right)$

A PPI-structure mapping distance matrix was constructed to provide dynamic features from two conditional probabilities (Supplementary Figure 1). From the column view, the matrix showed the spatial distance distribution of different amino acid residues ${PPI}_{j}$ in a protein structure $S_{i}$, $p\left( dis=i, {PPI}_{j} | S_{i} \right)$, which provided a ground static characteristic of distance distributions for each structure. From the row view, the matrix showed the distance distribution of different protein structures $S_{i}$ from the same interaction ${PPI}_{j}$, $p\left( dis=i, S_{i} | {PPI}_{j} \right)$, which provided a dynamic characteristic of distance changes within one PPI under different conditions. For both conditional probabilities, a high value implied a more easily captured mapping distance.

1. $\boldsymbol{p}\left( \boldsymbol{dis}_{\boldsymbol{min}}\boldsymbol{=i} \right)$

For each PPI-structure pair, the distance distribution of cross-linking sites generated by the homologous sequence in the same protein structure was fully considered, as well as the distance distribution of different structure states in the same protein structure (especially for NMR-generated structure). In previous applications, the minimum distances of these conditions were adopted as the actual distance of the interaction. By measuring the probability of minimum mapping distances, if a PPI exhibited a stable distance distribution in a homologous sequence or a different structural state, it increased the probability that ComMap recognizes it as a static interaction. Thus, a higher probability implied a more static distance.

$$p\left( {dis}_{min}=i \right)=\frac{\#(dis is min)}{\#(dis is min)+1}\cdot\cdot\cdot\cdot\cdot\cdot\cdot\cdot\cdot\cdot\cdot\cdot\cdot\cdot\cdot\cdot\cdot\cdot\cdot\cdot\cdot\cdot\cdot(1)$$

Where $\#(dis is min)$ represented the frequency of mapping distance $i$ as the minimum distance in one protein structure.

1. $\boldsymbol{DelDis}_{\boldsymbol{i}}$

The larger the mapping distance was generally considered to be the more dynamic the interaction. Therefore, the ${DelDis}_{i}$ was added to the scoring function for evaluation. Thus, a bigger ${DelDis}_{i}$value implied a more static distance.

$${DelDis}_{i}=\frac{1}{1+\left| {dis}_{i}- {dis}_{arm} \right|}\cdot\cdot\cdot\cdot\cdot\cdot\cdot\cdot\cdot\cdot\cdot\cdot\cdot\cdot\cdot\cdot\cdot\cdot\cdot\cdot\cdot\cdot\cdot(2)$$

Where ${dis}_{i}$ represented the mapping distance $i$, and ${dis}_{arm}$ represented the arm length of the cross-linker in the natural state.

1. $\boldsymbol{Com}\boldsymbol{Map Score}$

By integrating the above four factors. The ${PPI}_{j} S_{i} Score$ score was given to each PPI-Structure pair. The formula was shown below.

$${PPI}_{j} S_{k} Score=\frac{1}{p\left( dis=i, S_{k} | {PPI}_{j} \right)\times p\left( dis=i, {PPI}_{j} | S_{k} \right)\times p\left( {dis}_{min}=i \right)\times{DelDis}_{i}}\cdot\cdot\cdot\cdot(3)$$

Where ${PPI}_{j} S_{i} Score$ represented the score of PPI $j$ mapping protein structure $k$ with a distance $i$. Then, the score was normalized to get the $ComMap dynamic score$. During the normalization, PPI-structure pairs with outliers or too many missing values were filtered.

$$ComMap dynamic score=10\times\left( 1-\sin\left( \frac{\pi\times{PPI}_{j} S_{i} Score}{2\times max\left( {PPI}_{j} S_{i} Score \right)} \right) \right)\cdot\cdot\cdot\cdot\cdot\cdot\cdot\cdot\cdot\cdot\cdot\cdot\cdot\cdot\cdot\cdot\cdot\cdot\cdot(4)$$

For different protein structures of the same PPI, a wider distribution of scores with smaller values represented more dynamic interaction judgments, while a narrower distribution of scores with larger values represented more static judgments.

1. Results export

Finally, ComMap generated three files as outputs, including a comprehensive file that saved all PPI and structural distance information, a categorized file of PPIs based on structure entries, and a ComMap score file for PPI-structure. The categorized file offered pymol scripts for each protein structure for visualization. The ComMap score file provided ComMap scores for each PPI-structure, which reflects structural dynamism via a global view. For additional information, please check the user manual.

**References:**

Altschul, S.F.*, et al.* Basic local alignment search tool. *J Mol Biol* 1990;215(3):403-410.

Chen, Z.-L.*, et al.* A high-speed search engine pLink 2 with systematic evaluation for proteome-scale identification of cross-linked peptides. *Nat Commun* 2019;10(1):3404.

Harris, C.R.*, et al.* Array programming with NumPy. *Nature* 2020;585(7825):357-362.

Zhang, W., *et al*. SpotLink enables sensitive and precise identification of site nonspecific cross-links at the proteome scale. Brief. Bioinform. 2022:bbac316.

**Supplementary Note 2. A step-by-step ComMap guide on analyzing the bovine serum albumin (BSA) dataset**

Here, we provided a step-by-step tutorial to demonstrate how to analyze protein-protein interactions (PPIs) structural mapping with ComMap. In this tutorial, we selected the cross-linking data of BSA protein as an illustration for analysis. BSA is a simple and well-studied protein with a relatively stable protein structure. The BSA cross-linking results were published from multiple laboratories (Iacobucci, et al., 2019). We have organized the results, which can be accessed on GitHub (<https://github.com/DICP1810/ComMap>).

1. Software preparation and environment configuration

Firstly, hardware environment preparation. Please verify that the hardware environment satisfies the following system requirements. CPU: Intel or AMD processor with 64-bit support; 2.3 GHz or faster processor with at least 8 cores is recommended. RAM: 16GB or higher is recommended. Hard drive: 5GB or higher is recommended, 50GB or higher for proteome scale analysis is recommended. Please ensure Internet access for online mode analysis.

Secondly, software environment preparation. Please verify that the software environment satisfies the following requirements. OS: Windows 10 or higher (x64) is recommended. Runtime environment: python 3.8. Runtime-dependent libraries: Numpy and Pandas. A simple configuration method is to install Anaconda (release tag: Anaconda3-2021.05-Windows-x86_64).

Thirdly, software preparation. ComMap is open-source and can be freely downloaded on GitHub (<https://github.com/DICP1810/ComMap/releases>). After downloading the source code, please unzip it in a suitable location.

1. ComMap parameters configuration
2. Run ComMap

Firstly, project dictionary preparation. Please create a new folder for saving the project and result files. Please do not put ComMap code files in the same directory as project files. Then please copy the “Configuration.commap” file from the ComMap directory to the project file directory.

Secondly, input file preparation. Prepare the fasta file used to identify cross-links and the identification results generated from the cross-linking software. As for this tutorial, please download the fasta file (Tutorial/BSA.fasta) and cross-links (Tutorial/BSA_PPIs.txt) result from GitHub, and then copy two files into the project dictionary created above.

Thirdly, configuration file modification. Open the “Configuration.commap” file in the project dictionary in a text editor (Such as Visual Studio Code) and edit its entries. As for this tutorial, please modify the items by the sheet below. Other parameters remain unchanged by default.

| **Entry Name** | **Value** |
| --- | --- |
| A3_PATH_FASTA | E.g.: D:\BSA_tutorial\BSA.fasta ^a^ |
| C60_CALCULATION_TYPE | 1 |
| C60_PDB_STRUCTURE_PATH | E.g.: D:\ComMap\Data ^b^ |
| E1_PATH_EXPORT | E.g.: D:\BSA_tutorial ^c^ |
| C61_INPUT_LINK_RESULT_TYPE | 0 |
| C61_INPUT_LINK_RESULT_FILE | E.g.: D:\BSA_tutorial\BSA_PPIs.txt ^d^ |

^a^ Please enter the absolute path of the “BSA.fasta” file from the project folder.

^b^ Please enter the absolute path of the “Data” folder from the ComMap code folder.

^c^ Please enter the absolute path of the project folder.

^d^ Please enter the absolute path of the “BSA_PPIs.txt” file from the project folder.

Fourthly, ComMap analysis. Open a command line window and run ComMap as follows:

python [COMMAP_SCRIPT_LOCATION] [COMMAP_FILE_LOCATION]

COMMAP_SCRIPT_LOCATION: The location of the “ComMap.py” file in the ComMap directory. If you are unfamiliar with command operation, please filled with the absolute path of “ComMap.py”.

COMMAP_FILE_LOCATION: The location of the “Configuration.commap” file in the project directory. If you are unfamiliar with command operation, please fill with the absolute path of “Configuration.commap”.

After a short wait, ComMap reports the results. Supplementary Fig. 2A illustrates the log information during ComMap calculation. The logs generated during the actual process may differ from those shown in Supplementary Fig. 2A.

1. Result Analysis

The output results of ComMap can be found under the project folder, which is stored in three CSV files, “structure_alignment_result_total” file, “structure_alignment_result_PDB” file, and “structure_alignment_result_scored” file.

The “structure_alignment_result_total” file saved all PPI and structural mapping distance information. The “structure_alignment_result_PDB” file categorized all PPI structural mapping results based on protein structures. The “structure_alignment_result_PDB” file also included pymol scripts of each protein structure, which is convenient for visualization. As for this tutorial, a few BSA structures (PDB ID: 4OR0, 4F5S, and 6QS9), mapped with cross-links, visualized by pymol were illustrated in Supplementary Fig. 2B. The green line represents the interaction information within 30 angstroms, and the gray line represents the interaction information beyond 30 angstroms.

The “structure_alignment_result_scored” file contained the scoring information for the PPI-structure pair, which provided an indication of whether the PPI-structure is static or dynamic potentially. As for this tutorial, by evaluating the ComMap score distribution of ComMap, it was determined that most of the PPI-structure mappings were located in the high score partition, indicating that most BSA interactions are relatively static (Supplementary Fig. 2C).

**References:**

Iacobucci, C., *et al*. First Community-Wide, Comparative Cross-Linking Mass Spectrometry Study. *Anal. Chem.* 2019;91(11):6953-6961.

Supplementary Note 3. ComMap analysis on the proteasome dataset

To demonstrate the utility of ComMap PPI-structural analysis on targeted protein complexes, we analyzed the proteasome dataset. First, we studied the data from a global perspective and discovered that the ComMap results were not only rich but also conformed to fundamental cross-linking principles. Second, we analyzed the structural matching of proteasomes, demonstrating that ComMap can easily achieve high-density mapping. Third, we discussed the rationality of ComMap scoring and illustrated static and dynamic proteasome interactions using ComMap scores.

1. Global overview

As a brief introduction, this dataset was produced by BS3 cross-linked purified *Saccharomyces cerevisiae* 26S protein proteasome, downloaded from PXD011296 (Mintseris and Gygi, 2020).

By identifying cross-links with pLink2, a total of 2,685 PPIs were obtained at 1% FDR. ComMap analysis of these PPIs resulted in the mapping of 616 PPIs with 666 protein structures in the PDB database. A total number of 15,157 PPI-structure pairs were calculated.

The ComMap profiling was consistent with the basic foundations of cross-linking in that most cross-links were conservative (Ding, et al., 2017). From the distance distribution, most of the cross-linking information is located around 16 Å (Supplementary Fig. 3A), which matched the arm length of the BS3 cross-linker agent in its natural state. In addition, this conclusion was also supported by the distribution of ComMap scores, which suggests that most of the PPI-structure scores are located around the high score region (Fig. 1B).

1. High density structural mapping

ComMap can be used to obtain high-density PPI-structure mapping information. Many proteasome structures could map a large number of interactions, and ComMap enabled the visualization of protein complexes from cross-linking results clearly (Supplementary Fig. 3B). For example, two cryogenic electron microscopy (cryo-EM) structures of the proteasome, 6FVU and 6J2Q were clearly displayed (Supplementary Fig. 4). In both models, the majority of the cross-linking information was within the generally acceptable length (<30 Å) and were generally considered static interactions that could be supported by following ComMap score analysis. In conclusion, ComMap could generate high-density structural mapping models, which became the basis for subsequent research on biological problems.

1. ComMap score analysis on proteasome complex

To further demonstrate the benefit of ComMap scores in analyzing dynamics features, we performed an in-depth analysis of the proteasome interactions. First, we analyzed the distance matrix of ComMap among several interactions and structures (Supplementary Fig. 5A). The color in the matrix represented the distance for a given PPI-structure pair. In the matrix, from top to bottom, the minimum ComMap scores of interactions grown demonstrated an increasing tendency of static PPIs, which could be supported by the color shift in the heatmap. In more detail, the upper half of the matrix exhibited a significant color change, whereas the lower half exhibited only a slight color change. The ComMap score, the change in color in the heat map, and the dynamical tendency of proteins all reflected the same trend. Thus, ComMap scores were reasonable indicators reflecting protein dynamical features intuitively.

Second, we illustrated several PPI-structure pairs based on the ComMap score. ComMap scores from Q06103 (104, Lys) - Q06103 (111, Lys) and Q12250 (141, Lys) - Q12250 (105, Lys) were consistent with 9.9, which reports a static interaction. All structures including the cryo-EM structures 6J2N and 3JCK provide support for this conclusion (Supplementary Fig. 5B). Additionally, ComMap scores from Q12377 (7, Lys) - Q12377 (49, Lys) spanned a wide range, reaching 3.1, and its lower limit score reached 3.5, which shows a dynamic interaction. The cryo-EM structures 4CR3 and 5MPC provide support for this conclusion (Supplementary Fig. 5C). Structure 5MPC was generated in the absence of beryllium fluoride (BeFx) (Wehmer, et al., 2017) ,and structure 4CR3 was acquired in the presence of ATP and ATP-γS (Unverdorben, et al., 2014). Under different surrounding environments, protein Q12377 (7, Lys – 49, Lys) exhibited different protein conformations.

ComMap captured dynamism features based on the existing structures and reported valuable scores, which could benefit biologists in future structural analysis and simulation.

**References:**

*Ding, Y.H., et al*. Modeling Protein Excited-state Structures from "Over-length" Chemical Cross-links. *J Biol Chem 2017;292(4):1187-1196.*

Mintseris, J. and Gygi, S.P. High-density chemical cross-linking for modeling protein interactions. *Proc Natl Acad Sci U S A* 2020;117(1):93-102.

Unverdorben, P.*, et al.* Deep classification of a large cryo-EM dataset defines the conformational landscape of the 26S proteasome. *Proc Natl Acad Sci U S A* 2014;111(15):5544-5549.

Wehmer, M.*, et al.* Structural insights into the functional cycle of the ATPase module of the 26S proteasome. *Proc Natl Acad Sci U S A* 2017;114(6):1305-1310.

Supplementary Note 4. ComMap analysis on the K562 cells dataset (proteome scale PPIs)

To demonstrate the capability of ComMap high-throughput PPI-structural analysis on proteome-scale protein complexes, we analyzed the K562 cells cross-linking dataset. First, we studied the ComMap results from a global perspective, the conclusion was similar to the proteasome dataset. Second, we analyzed the structural mapping of several complexes, demonstrating that ComMap can easily achieve high-throughput analysis. Third, we discussed the dynamical features of a few complexes based on ComMap. Fourth, we provided new insight into ribosome interactions by ComMap analysis.

1. Global overview

As a brief introduction, this dataset was produced by DSSO cross-linked human K562 cells, downloaded from PXD08771 (Yugandhar, et al., 2020).

By identifying cross-links with XLinkX, a total of 14495 PPIs were obtained at 1% FDR. ComMap analysis of these PPIs resulted in the mapping of 2,651 PPIs with 8284 protein structures in the PDB database. A total number of 30,7034 PPI-structure pairs were calculated. ComMap finished the analysis within one hour on a general 64 GB RAM workstation.

The ComMap profiling was consistent with the basic foundations of cross-linking in that most cross-links were conservative. From the distance distribution, it is shown that most of the cross-linking information is located around 18 Å (Supplementary Fig. 6A), which corresponds to the arm length of the DSSO cross-linker in its natural state. In addition, this conclusion was also supported by the distribution of ComMap scores, which suggests that most of the PPI-structure scores are located around the high score region (Fig. 1B).

1. High density structural mapping

High-density PPI-structure information can be obtained through the ComMap mapping process. Many protein structures can match a large amount of interaction information (Supplementary Fig. 6B-6H), including ribosome-related protein complexes with the largest number of matches, followed by heat shock-related proteins and several mitochondrial complexes. The high-throughput mapping of ComMap provided a powerful tool for the cross-linking analysis of multiple protein complexes within the same cellular or sub-cellular spacetime.

1. ComMap scores analysis on few complexes

Next, we illustrated several dynamical interactions based on ComMap scores.

P62979 is a ubiquitin protein present in 40S ribosomes. ComMap reported its spatial conformational changes when it interacts with methyltransferase and E3 ubiquitin-protein from structures 6KIV and 5YDK. ComMap scores of P62979 (33, Lys) - P62979 (48, Lys) span a wide range reaching 7.7, and its lower limit score hits 1.9 (Supplementary Fig. 7A).

P62826 is GTP-binding nuclear protein, involved in nucleocytoplasmic transport, participating both in the import and export from the nucleus of proteins and RNAs. ComMap reported its spatial conformational changes when it forms complex with Pdr6-eIF5A and Nup153ZnF1 from structures 6Q84 and 3GJ6. ComMap scores of P62826 (37, Lys) - P62826 (60, Lys) span a wide range reaching 8.6, and its lower limit score hits 1.0 (Supplementary Fig. 7B).

P0DP23 is a calmodulin protein, control by of a large number of enzymes, ion channels, aquaporins, and other proteins through calcium binding. ComMap reported its spatial conformational changes when it forms complex with lens fiber major intrinsic protein and voltage-gated channel subfamily KQT protein from structures 3J41 and 6FEH. ComMap scores of P0DP23 (78, Lys) - P62979 (149, Lys) span a wide range reaching 5.9, and its lower limit score hits 3.9 (Supplementary Fig. 7C).

1. New insights on ribosome complex

Finally, we analyzed the interactions between 80S ribosomal proteins (Klinge and Woolford, 2019). The two subunits of 80S ribosomes, 40S ribosomes, and 60S ribosomes, played an important role in the protein translation process (Baßler and Hurt, 2019). From the ComMap analysis, we found a large number of potential dynamic interactions (Supplementary Fig. 8A). ComMap scores distribution revealed that most of the scores between the 40S and 60S were relatively low (6 to 8) and spanned a wide range, indicating highly dynamical features (Supplementary Fig. 8B). By matching these interactions with the known structure 6IP8, the number of interactions over 100 Å (37) is greater than the number of interactions within 100 Å (4) (Supplementary Fig. 8C). Other protein structures, such as 6ZMI, 4UG0, 6Y0G, also reported similar distance distributions. The results indicated that current structures are incapable of explaining the dynamics of the interaction between the 40S and 60S, and ComMap scores implied the existence of more dynamic structures. As a prospect, ComMap analysis may provide more information for the simulation from PPIs to structures of protein complexes, including ribosomes.

**References:**

Baßler, J. and Hurt, E. Eukaryotic Ribosome Assembly. *Annual Review of Biochemistry* 2019;88(1):281-306.

Klinge, S. and Woolford, J.L. Ribosome assembly coming into focus. *Nature Reviews Molecular Cell Biology* 2019;20(2):116-131.

Yugandhar, K.*, et al.* Structure-based validation can drastically underestimate error rate in proteome-wide cross-linking mass spectrometry studies. *Nat. Methods* 2020;17(10):985-988.


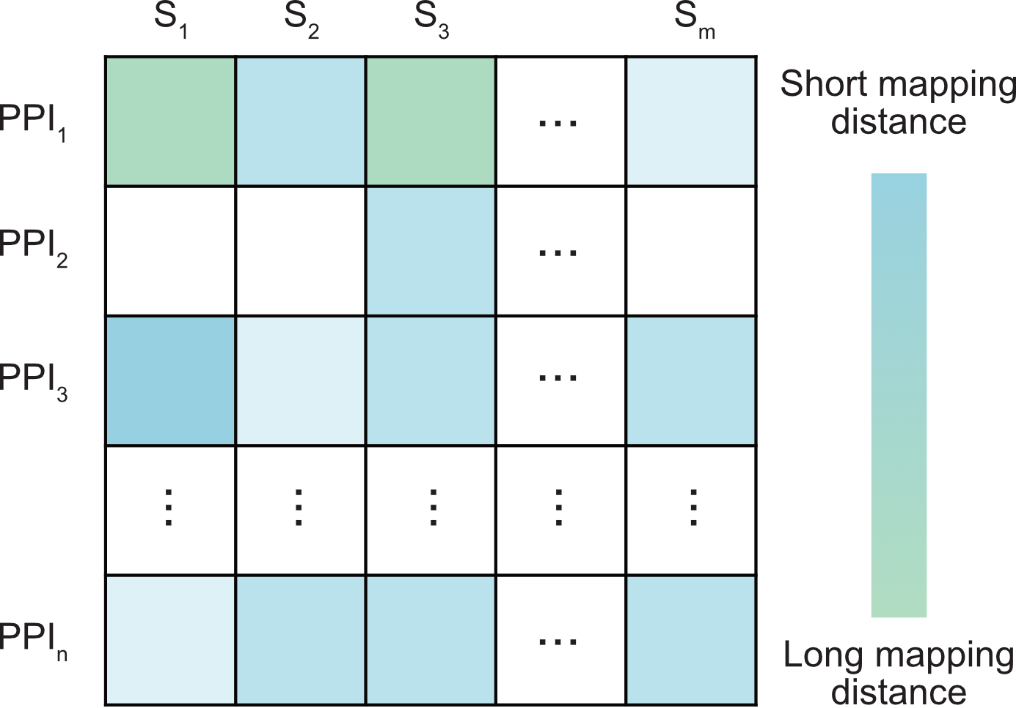


**Supplementary Figure 1. The PPI-structure matrix is used to score and rank in ComMap.** The size of the matrix is $n\times m$, where $n$ represents the total number of protein-protein interactions and $m$ represents the total number of mapping protein structures. The color in the block represents the length of the mapping distance. White blocks represent the missing values.


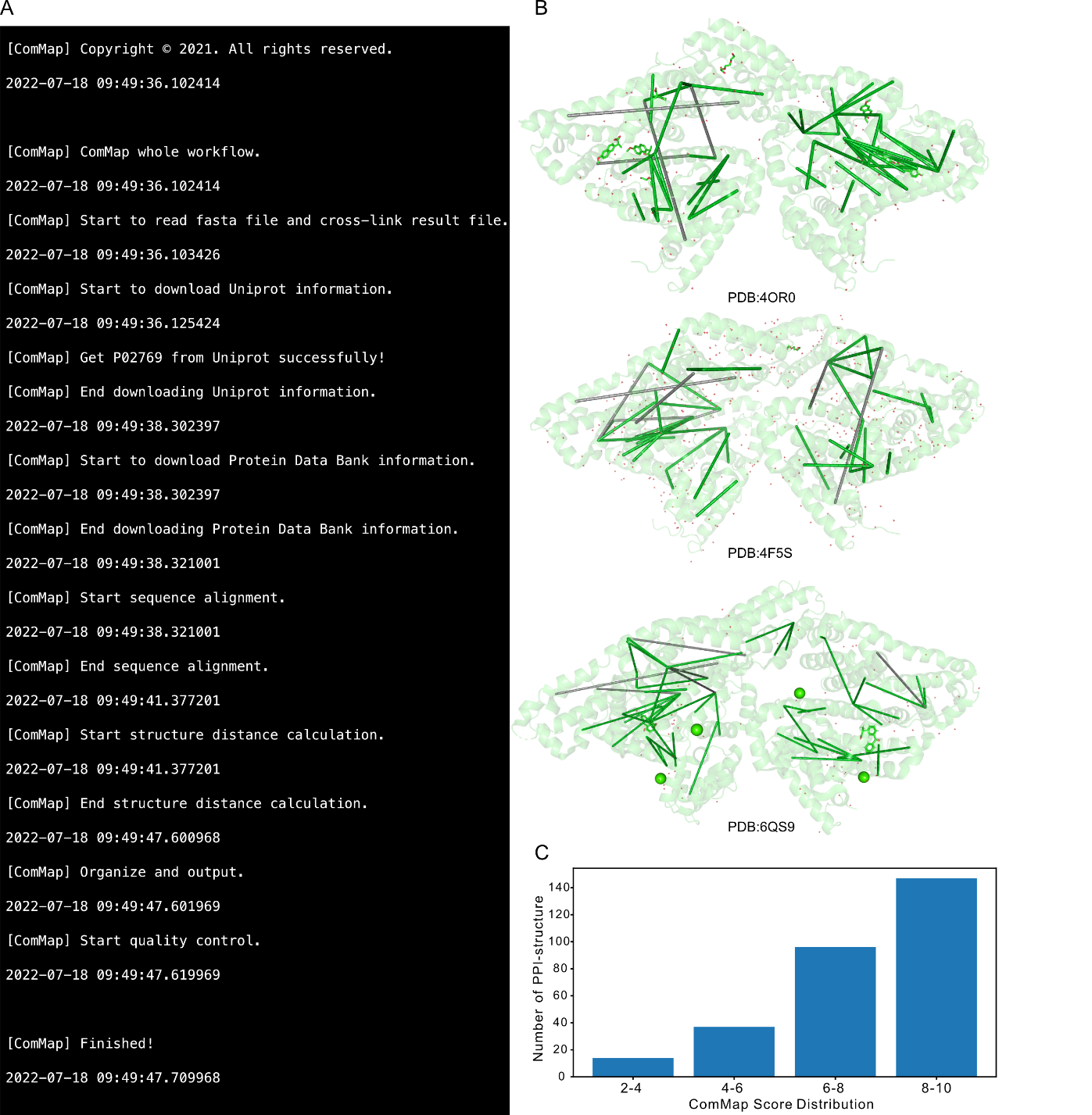


**Supplementary Figure 2. ComMap guide on analyzing the bovine serum albumin (BSA) dataset.** (A) The log information during ComMap analysis. (B) An illustration of BSA structures (PDB ID: 4OR0, 4F5S, and 6QS9) mapping with cross-links. The green line represents the interaction information within 30 angstroms, and the gray line represents the interaction information beyond 30 angstroms. (C) The ComMap score distribution of matched the PPI-structure.


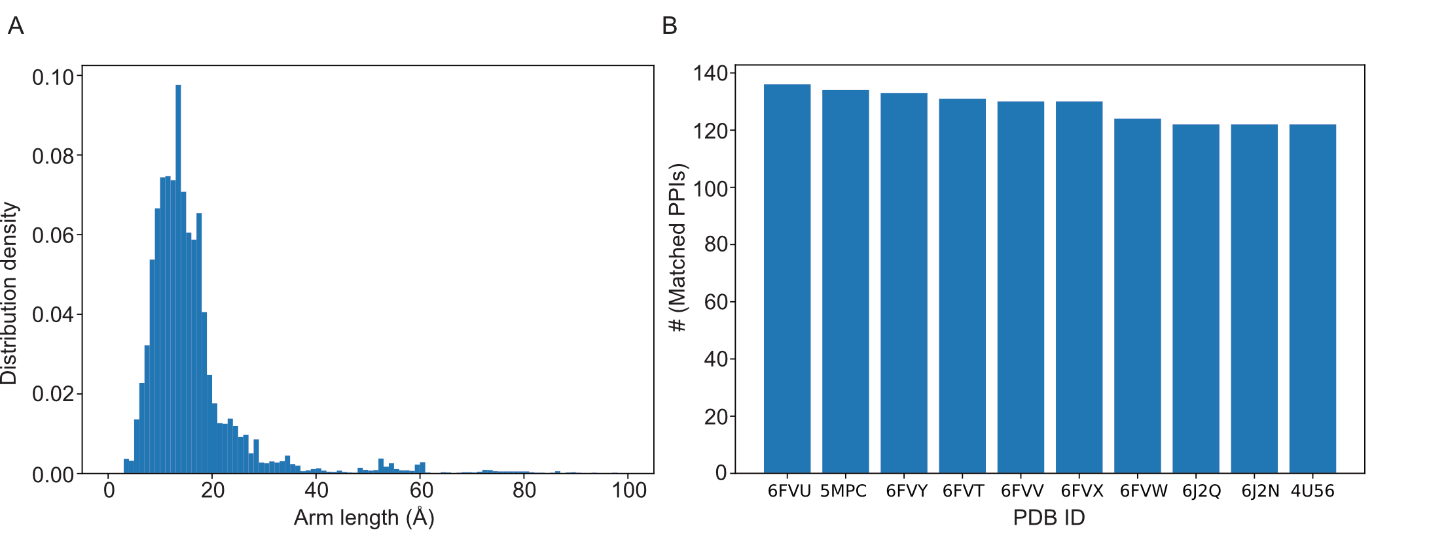


**Supplementary Figure 3. The overall PPI-structure matching of ComMap from PXD011296.** (A) The Cα-Cα distance distribution of all PPI-structure matches. (B) The top 10 matched PDB structures with the most abundant of PPIs.


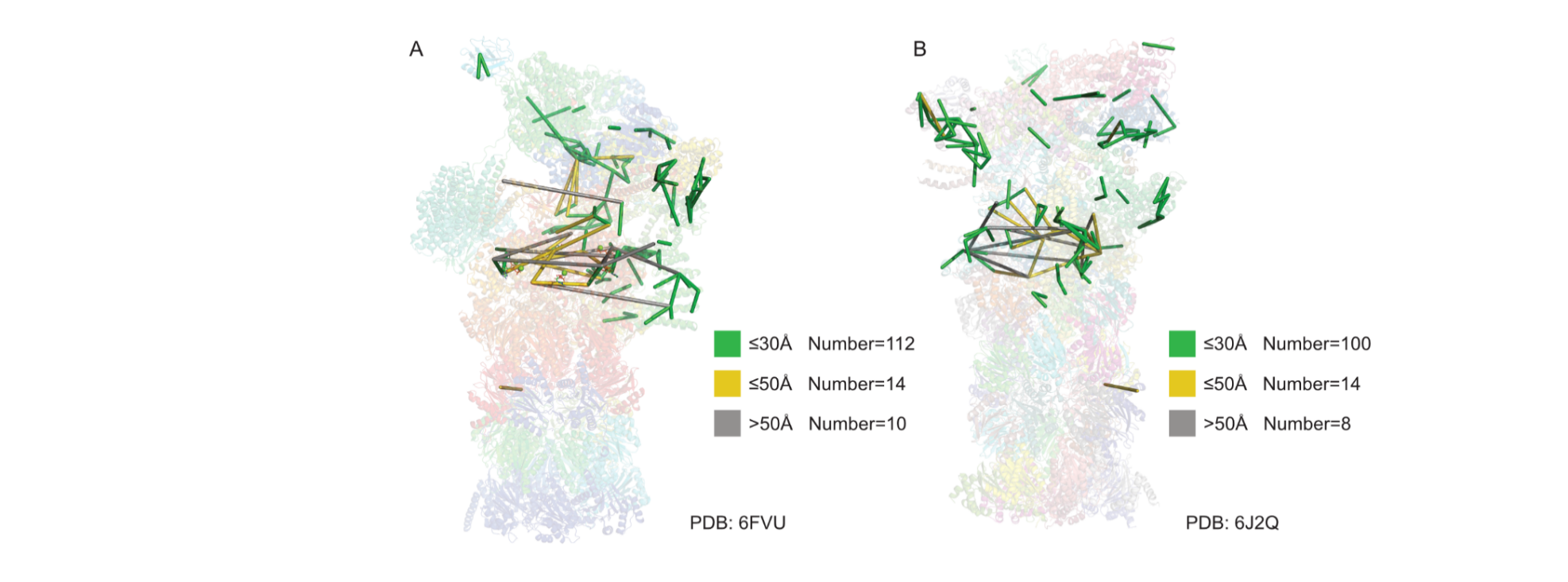


**Supplementary Figure 4. Two examples of structure matching from proteasome complex.** The visualization of structure matching can be easily completed by the pymol script from ComMap output results. (A) The structure 6FVU, which represented the proteasome in s2 state. (B) The structure 6J2Q, which represented the proteasome in ubiquitin-accepted state.


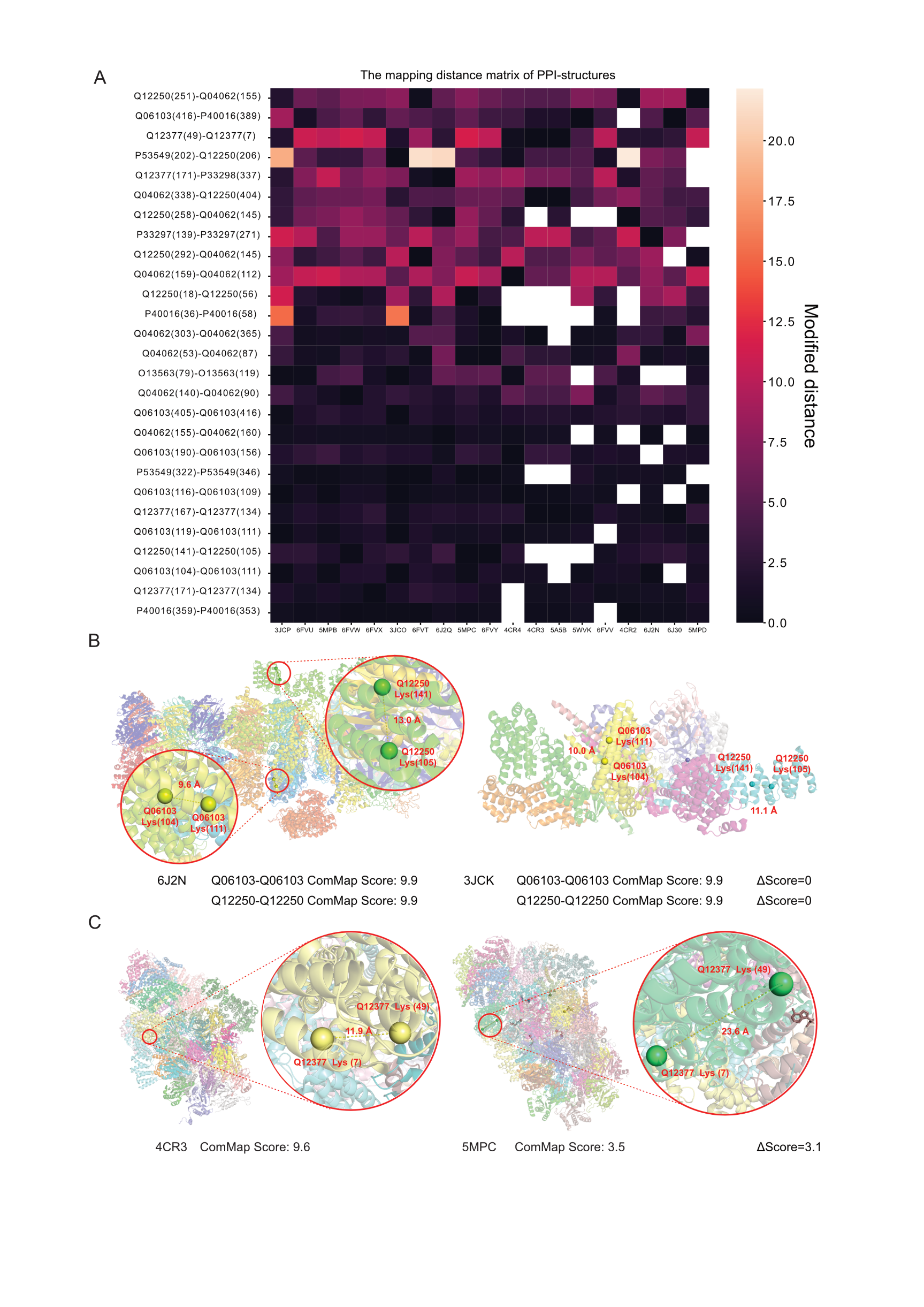


**Supplementary Figure 5. ComMap score analysis on proteasome dataset.** (A) An illustration of PPI-structure distance matrix for some proteasome cross-links. Each row represented a PPI. Each column represented a PDB structure. The color represented the modified distance for a given PPI-structure pair. The modified distance was calculated by subtracting the minimum value of each row from the original distance. The white blocks in the matrix represented missing values. The matrix was ordered from top to bottom in increasing order of ComMap score. (B) An illustration of dynamic interactions reported by ComMap score. (C) An illustration of dynamic interaction reported by ComMap.


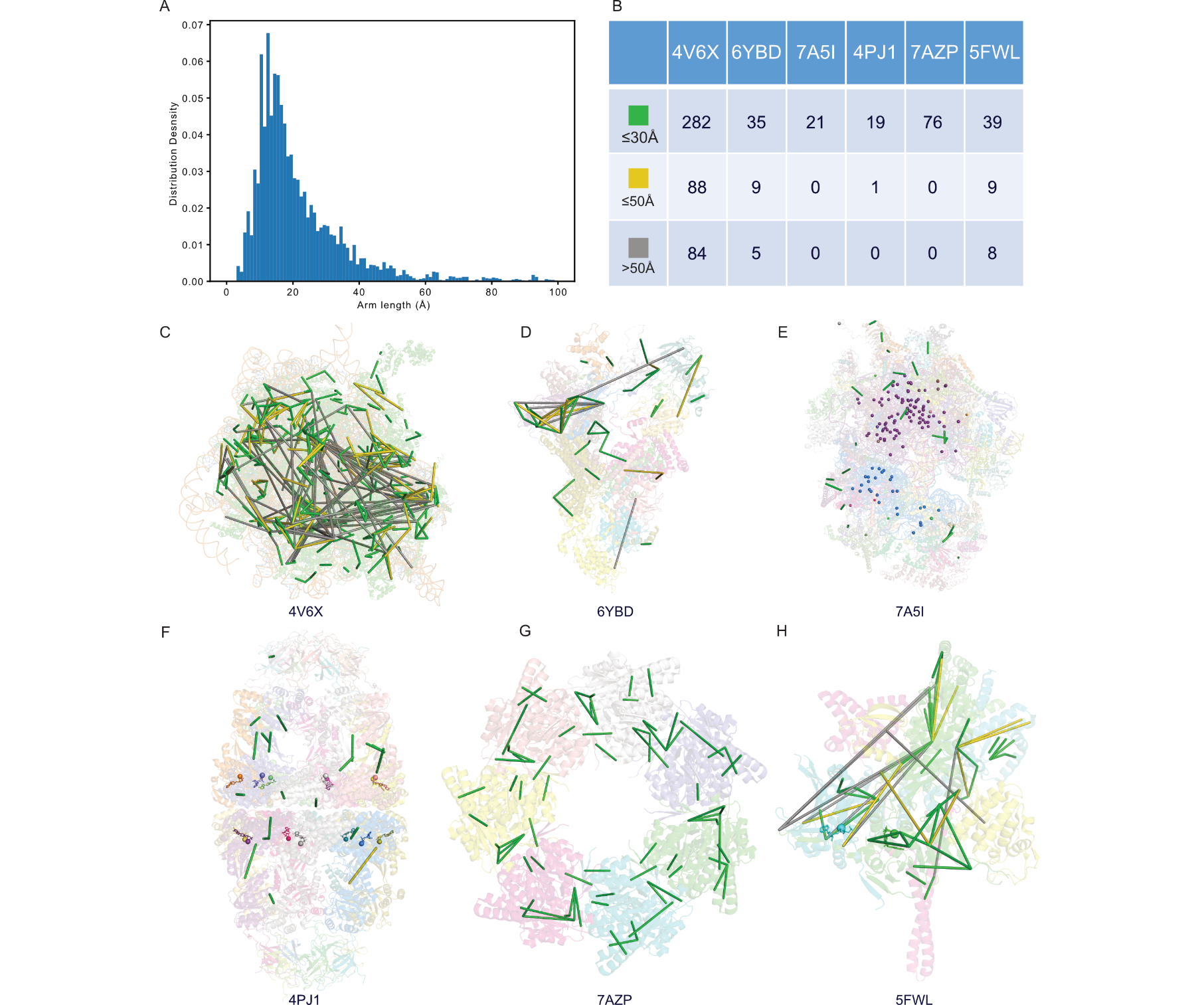


Supplementary Figure 6. The ComMap mapping results of the K562 cells dataset. (A) The Cα-Cα distance distribution of all PPI-structure matches. (B) The sheet of mapping distance-PPI counts from several structures in (C)-(H). (C) The PPI matching of human 80S ribosomal on 4V6X. (D) The PPI matching of human 48S translational initiation complex on 6YBD. (E) The PPI matching of human mitoribosome on 7A5I. (F) The PPI matching of human mitochondrial chaperonin symmetrical 'football' complex on 4PJ1 (G) The PPI matching of human mitochondrial HSPD1 single ring on 7AZP. (H) The PPI matching of Hsp90-Cdc37-Cdk4 complex on 5FWL.


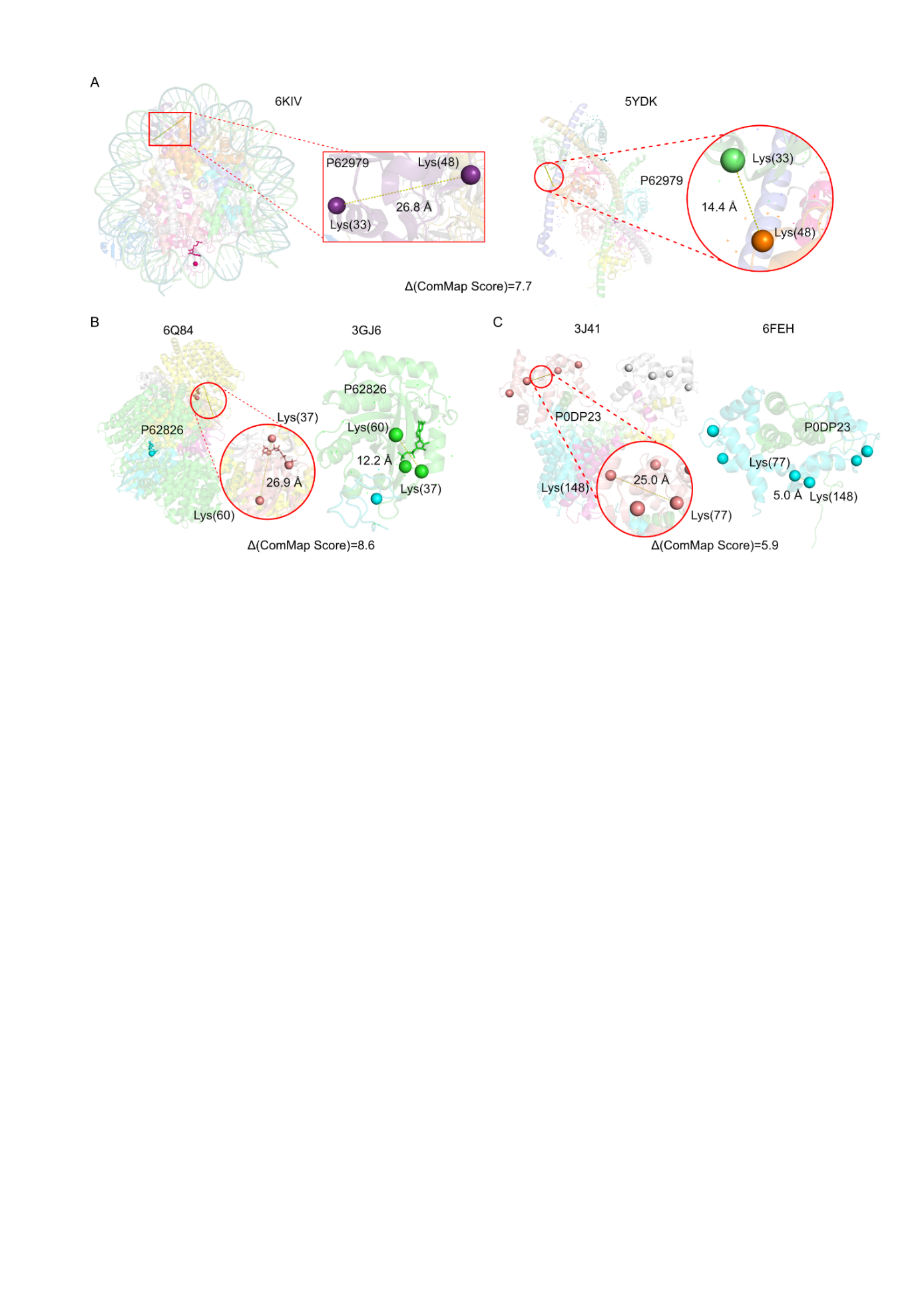


Supplementary Figure 7. Several dynamic interactions of protein complexes reported by ComMap. (A) The dynamic PPI of P62979 from structures 6KIV and 5YDK. (B) The dynamic PPI of P62826 from structures 6Q84 and 3GJ6. (C) The dynamic PPI of P0DP23 from structure 3J41 and 6FEH.


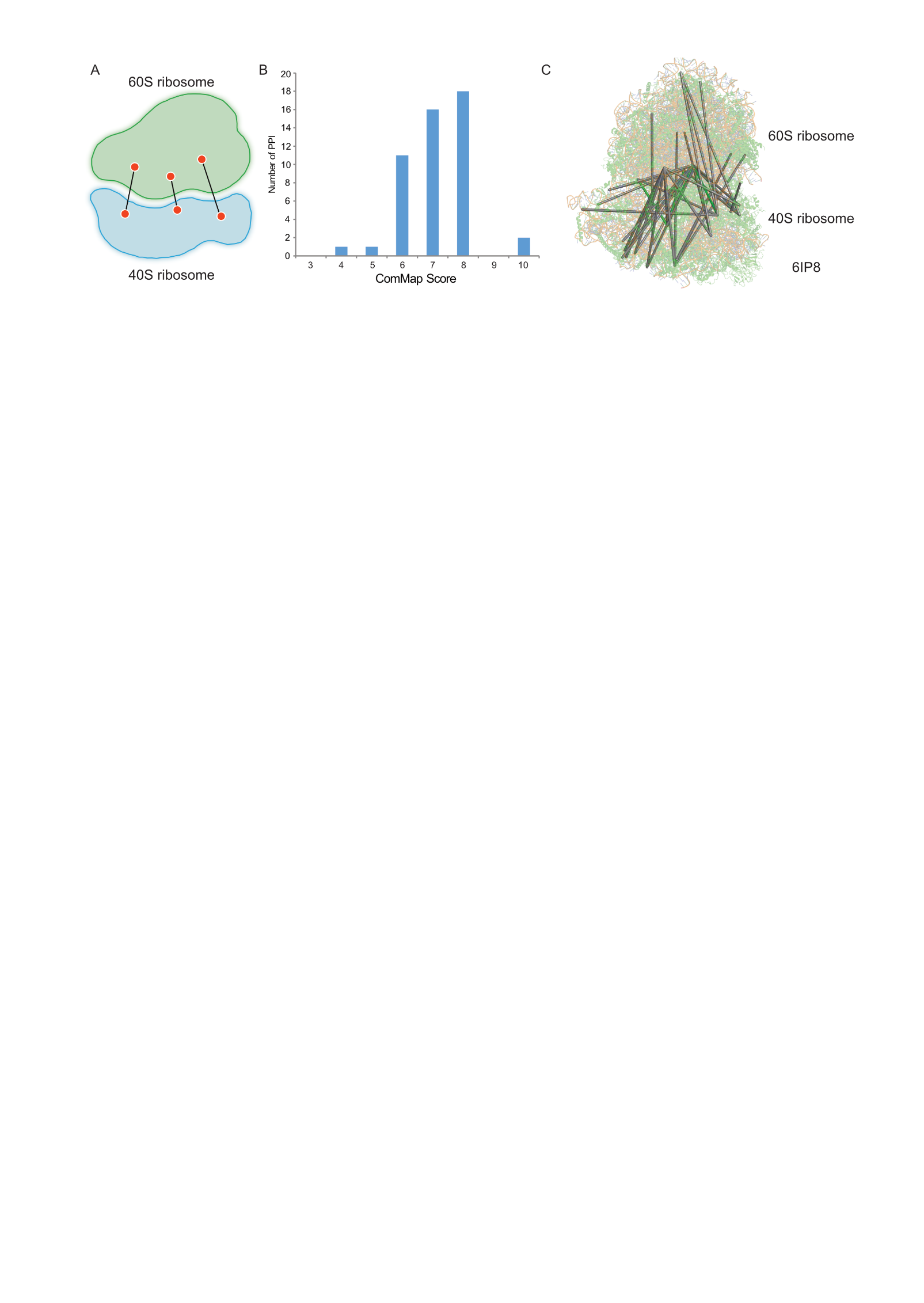


Supplementary Figure 8. The ComMap study of PPIs between 40S ribosome and 60S ribosome. (A) The interactions between 40S ribosome and 60S ribosome. (B) The ComMap score distribution between the 40S subunit and the 60S subunit. (C) PPIs between the 40S subunit and the 60S subunit matching on PDB structure 6IP8, the green line represents distance less than 100 Å, the grey line represents distance more than 100 Å.

Altschul, S.F.*, et al.* Basic local alignment search tool. *J. Mol. Biol.* 1990;215(3):403-410.

Baßler, J. and Hurt, E. Eukaryotic Ribosome Assembly. *Annual Review of Biochemistry* 2019;88(1):281-306.

Chen, Z.-L.*, et al.* A high-speed search engine pLink 2 with systematic evaluation for proteome-scale identification of cross-linked peptides. *Nat. Commun.* 2019;10(1):3404.

Ding, Y.H.*, et al.* Modeling Protein Excited-state Structures from "Over-length" Chemical Cross-links. *J. Biol. Chem.* 2017;292(4):1187-1196.

Harris, C.R.*, et al.* Array programming with NumPy. *Nature* 2020;585(7825):357-362.

Iacobucci, C.*, et al.* First Community-Wide, Comparative Cross-Linking Mass Spectrometry Study. *Anal. Chem.* 2019;91(11):6953-6961.

Klinge, S. and Woolford, J.L. Ribosome assembly coming into focus. *Nature Reviews Molecular Cell Biology* 2019;20(2):116-131.

Mintseris, J. and Gygi, S.P. High-density chemical cross-linking for modeling protein interactions. *Proc. Natl. Acad. Sci.* 2020;117(1):93-102.

Unverdorben, P.*, et al.* Deep classification of a large cryo-EM dataset defines the conformational landscape of the 26S proteasome. *Proc. Natl. Acad. Sci. U. S. A.* 2014;111(15):5544-5549.

Wehmer, M.*, et al.* Structural insights into the functional cycle of the ATPase module of the 26S proteasome. *Proc. Natl. Acad. Sci. U. S. A.* 2017;114(6):1305-1310.

Yugandhar, K.*, et al.* Structure-based validation can drastically underestimate error rate in proteome-wide cross-linking mass spectrometry studies. *Nat. Methods* 2020;17(10):985-988.

Zhang, W.*, et al.* SpotLink enables sensitive and precise identification of site nonspecific cross-links at the proteome scale. *Brief. Bioinform.* 2022:bbac316.
